# Difficult decisions in pediatric practice and moral distress in the intensive care unit

**DOI:** 10.5935/0103-507X.20180039

**Published:** 2018

**Authors:** Raissa Passos dos Santos, Daniel Garros, Franco Carnevale

**Affiliations:** 1 Ingram School of Nursing, McGill University - Montréal, Quebec, Canada.; 2 Stollery Children's Hospital - Edmonton, Alberta, Canada.

**Keywords:** Ethics, clinical, Morals, Stress, psychological, Conflict (psychology), Decision making/ethics, Attitudes of health personnel, Infant, Child, Intensive care units, pediatric/ethics

## Abstract

In an ethical dilemma, there is always an option that can be identified as the best one to be chosen. When it is impossible to adopt such option, the situation can lead professionals to experience moral distress. This review aims to define the issue of moral distress and propose coping strategies. Systematic searches in the MEDLINE/PubMed and SciELO databases were conducted using the keywords "moral distress" and "moral suffering" in articles published between 2000 and 2017. This review was non-exhaustive and contextual, with a focus on definitions, etiologies and methods of resolution for moral distress. In the daily practice of intensive care, moral distress was commonly related to the prolongation of patients' suffering and feelings of helplessness, as well as difficulties in communication among team members. Coping strategies for moral distress included organizational, personal and administrative actions. Actions such as workload management, mutual support among professionals and the development of techniques to cultivate open communication, reflection and questioning within the multidisciplinary team were identified. In clinical practice, health professionals need to be recognized as moral agents, and the development of moral courage was considered helpful to overcome ethical dilemmas and interprofessional conflicts. Both in pediatric and adult intensive care, professionals are challenged by questions about their practice, and they may experience moral distress. This suffering can be minimized and solved by understanding that the focus is always on the patient and acting with moral courage and good communication in an environment of mutual respect.

## INTRODUCTION

### Ethical considerations in pediatric intensive care: fundamental grounds

The current model of care for seriously ill patients has particular complexities for health teams.^(^^1^^)^ In pediatric practice, pediatric intensive care units (ICU) are environments with high occupational stress. The highly technological work environment, the demands of aggressive procedures and the uncertainty inherent in the prognoses result in high psychological pressure for professionals. Additionally, factors such as a lack of hospital beds, the need to work as a team and family involvement in decision making contribute to this challenging environment.^(^^2^^)^ Reports from a multidisciplinary study conducted in six Canadian ICUs have shown that witnessing the suffering of a child and his or her family weakens professionals. These situations worsen when the ICU's structure and decision-making process are fragmented and when there is a lack of consensus among the parties involved in these scenarios.^(^^3^^)^

In the trajectory of the child hospitalized in the pediatric ICU, other factors are considered extremely stressful for the health team. These include the imposition of a treatment because of pressure from parents and/or guardians, performing painful and sometimes unnecessary procedures, participation in treatments that extend the suffering or death process of the child, interactions with professionals with little experience and conflicts between team members. These situations become triggers for distress and suffering for the professionals involved^(^^1^^)^ and characterize what the literature currently describes as "moral distress."^(^^4^^-^^6^^)^

Thus, the purpose of this article was to review the literature to describe the phenomenon of moral distress within pediatric intensive care practice and to determine its possible causes. Another goal was to propose coping strategies at the organizational, personal and administrative levels in order to minimize this problem.

To achieve these goals, articles were selected using the authors' expertise to search the MEDLINE/PubMed and SciELO databases and to perform snowball sampling with a focus on definitions, etiologies and therapeutics. Articles published between 2000 and 2017 were included in the search due to the increase in the number of articles published since 2000. We analyzed 25 articles and 1 master's dissertation. Only two studies presented Brazilian data.

### Moral distress: barriers to ethical action in pediatric intensive care

The phenomenon of moral distress was first defined by Jameton in 1984 as painful feelings experienced by a professional resulting from the impossibility of acting in the way he or she considers correct.^(^^7^^)^ Intrinsic sources (personal values) or extrinsic sources (barriers in the environment, such as in the institution or workplace) can determine this suffering, causing the professional to be prevented from acting in the way he or she considers correct.^(^^7^^)^ It is therefore a personal perception: "I know what I should do, but I cannot do it." Feelings related to moral distress may include anger, guilt, frustration and hopelessness, and these may manifest as physical reactions such as muscle pain, diarrhea, sleep disturbances and fatigue.^(^^7^^,^^8^^)^

In the time since the concept was defined, several studies have tried to identify and measure moral distress in health teams. Corley et al. developed and validated a scale that describes the frequency and intensity of moral distress in various healthcare scenarios. The scale has 32 items that reflect moral distress in the work of nurses, with levels ranging from little (1) to intense (5).^(^^9^^)^ The scale was further simplified and adapted by Hamric et al. to suit different specialties, including pediatrics, adult patient care and other health professional fields.^(^^10^^)^

The use of this concept was first related to nursing practice in a hospital context and described suffering resulting from power and hierarchy relations found in the profession and from nurses' senses of obligation to advocate for patients, among other issues.^(^^7^^)^ Recently, research has evaluated this phenomenon in other health professionals.^(^^2^^,^^11^^)^ In a study involving approximately 2,000 professionals, moral distress was observed in physicians, nurses, pharmacists, occupational therapists, nutritionists, speech therapists, physical therapists, social workers and chaplains.^(^^12^^)^

Work done with doctors and nurses in adult ICUs revealed institutional context as one of the factors that triggers moral distress.^(^^8^^)^ Examples include a scarcity of resources for adequate patient care and a need to promote less-than-ideal care due to the imposition of cost-cutting measures by administrators. Still, the need to provide care that (falsely) increases the hopes of the family or persistence in a treatment that prolongs life exclusively based on the desire and expectation of the family were factors reported as more relevant. In these cases, the phenomenon of moral distress is directly related to professional dissatisfaction and may determine the desire to leave the profession.^(^^8^^)^

A study conducted in pediatric and neonatal ICU in Toronto, Canada, with 89% participation, showed that 58% of professionals (physicians, nurses and respiratory therapists) reported moral distress related to their work. The most relevant causative factors were related to end-of-life care and communication. "Accepting the wishes of the family and continuing life support even when I believe it is not best for the child" was the most commonly mentioned element.^(^^13^^)^

The intensity of a clinical encounter that triggers moral distress is more important than the frequency of these cases. This was observed in studies of both adult and pediatric ICU.^(^^8^^,^^13^^)^ Table 1 presents the main causes of moral distress recorded in the literature.

**Table 1:** Main causes of moral distress

Clinical situations	Internal barriers	External barriers
Providing unnecessary/futile treatment	Feeling helpless	Inadequate communication between team members
Prolonging the death process through aggressive treatment	Inability to identify ethical issues	Divergent inter- (doctor and nurse) and intraprofessional (nurse and nurse) perspectives
Inadequate patient informed consent	Lack of understanding about the situation	Insufficient number of professionals and increased workload
Working with professionals who are not competent	Self-doubt	Lack of administrative support
Lack of consensus on treatments	Lack of knowledge about alternative treatment plans	Policies and priorities conflicting with care needs
Lack of continuity of care	Greater moral sensitivity	Following the wishes of family members for fear of legal proceedings
Inappropriate use of resources	Lack of assertiveness	Tolerance of abusive behavior
Providing care in disagreement with the best interests of the patient	Socialization to follow others	Compromising care due to cost-cutting measures
Providing inadequate pain relief		Hierarchies within the health system
Providing false hope to patients and families		Lack of professional teamwork
Accelerating the death process		Nurses not involved in the decision-making process
Lying		Commitment to care due to health insurance pressure and fear of legal proceedings
Disregarding the wishes of the patient		

Source: Adapted from Hamric AB, Borchers CT, Epstein EG. Development and testing of an instrument to measure moral distress in healthcare professionals. AJOB Prim Res. 2012;3(2):1-9.^(^^10^^)^

Thus, it becomes important to differentiate moral distress from ethical dilemmas. In the case of a dilemma, team members can express different and perfectly acceptable opinions about which treatment to follow.^(^^7^^,^^14^^)^ However, depending on how the case is conducted, some professionals may experience moral distress. This occurs from the feeling of being involved in, witnessing or taking part in something "wrong" when your preferred option was not adopted.^(^^14^^)^

### Principle of "the child's best interest": "if the child could speak, what would she choose..."

In regard to ethical dilemmas and moral distress, one cannot omit the fact that decision-making in pediatric care is complex because it involves intermediaries or *proxies* - parents or guardians. To this end, the principle most accepted worldwide for decision-making is based on what is known as the child's "best interest," which is expressed in Article 227 of the Brazilian Federal Constitution.

The use of this concept involves the premise that any decision involving the health of the child should be the one in which the benefits to the child outweigh the potential harm and in which the focus is on the child and his or her well-being rather than that of the family or guardians.^(^^15^^,^^16^^)^ The lack of homogeneity for the definition of what the "best interests" of the child are, in fact, is a limiting factor for its use in clinical practice. This occurs because a convergence of beliefs and values between the team, family and child is not always possible.^(^^16^^)^ In addition, difficulties in the physician-nurse relationship, professionals' concerns about how to consider the child's voice in the decision-making process and fears of making mistakes, in addition to the need to take care of the family as a whole, add to the complications in this matter.^(^^15^^,^^16^^)^ In this type of situation, feelings of doubt about the correct conduct can result in moral distress for professionals.^(^^15^^,^^16^^)^

### Principals of engagement and moral agent in pediatric intensive care: "it is the price to pay"

In addition to having the ability to perceive the phenomenon of moral distress, it is important to also uncover how it occurs. Understanding the phenomena of engagement and moral agency is essential to addressing moral distress and improving the decision-making process using the principle of "best interests."

Questions about how pediatric ICU professionals do or do not engage in various care situations are important in recognizing this ethical dimension of daily practice. In a recent study of nurses in pediatric units, several levels of engagement were found.^(^^17^^)^ This means that in cases where the child needs a painful procedure, for example, a highly engaged nurse may understand this situation as difficult because of feelings of guilt and frustration over performing an action that causes the child pain. Consequently, he or she experiences moral distress due to taking an action that, in his or her perception, harms the child. However, a less engaged professional may understand pain in the severely ill child as unavoidable (inherent in the care and as a consequence of the illness) and thus disconnect from the results of his or her actions, thereby preventing, in a way, moral distress.^(^^17^^)^

This also applies to the physician, who can minimize the situation by using defenses that make him or her understand the situation as unavoidable within the context of the illness, that is, "the price to be paid." However, the professional does not fail to recognize the trauma that the illness represents for the family and the child.^(^^17^^)^ Studies report that when disengaging from situations, professionals seek justifications in their attitudes that minimize and disconnect them from the consequences of actions that may be considered unethical.^(^^18^^,^^19^^)^ However, the mechanisms that make professionals more or less engaged are still not well described in the literature.

In this way, health professionals are expected to be morally engaged in their practice so that their actions are morally in the best interests of the child.^(^^20^^)^ Still, engagement must be promoted in health services so that ethical practices can be achieved. To do this, one must move from the reality of moral distress to a reality in which ICU team members carry out their agency functions and advocate for patients. The concept of "agency," first described in philosophy, refers to a person's ability to engage meaningfully with a particular situation. More specifically, moral agency refers to the individual's ability to engage in morally accentuated or highly impacting situations. That is, situations that fall into a dichotomy spectrum of good or bad, right or wrong, and fair or unfair.^(^^20^^)^

### Recognition of professionals in intensive care as moral agents: "there is no other way" and "let us do this so that everything will be all right"

Moral agency plays a fundamental role in enriching the ethical practice of health professionals in the ICU. Only using of the concept of moral distress, which is extremely important for understanding professional ethical practice, can lead to a limited understanding of this complex dimension and leave the professional in a situation of conformity - "there is no other way." This is because by using it alone, the health professional is limited to being in the position of a victim, leading to a minimization of reality and disregard for the role of engagement in the dilemmas experienced in daily practice.^(^^17^^,^^20^^)^

"Moral agents" can be defined as professionals who strive to find the right attitude to be taken. More specifically, the literature describes the role of a moral agent as having moral courage in practice.^(^^21^^)^ Moral courage can be defined as "an individual's ability to overcome fear and to fight for the values in which he or she believes, which he or she considers fundamental." It is the will to speak and do what is right in the face of forces that would lead a person to act in some other way without being intimidated. He or she puts "principles into action."^(^^21^^)^

In ethical practice, moral courage is the connection between the recognition by the professional of the correct attitude to be taken and the consequent accomplishment of this action.^(^^21^^,^^22^^)^ That is, reflection on the correct attitude to be taken becomes moral courage when it is activated by conduct on behalf of the patient according to personal obligations and values. However, proposing behaviors, taking action and exercising moral courage can bring negative consequences and risks to the professional, such as conflicts with colleagues, humiliation, rejection and ridicule.^(^^21^^)^ Factors that inhibit or attempt to stifle moral courage include organizational cultures that stifle discussions of unethical behavior, willingness to compromise personal standards and professional codes in order to avoid social isolation ("let us do this so everything will be all right") or to ensure a promotion or favoritism within the organization, difficulty in confronting unethical behavior, indifference to ethical values, apathy of professionals due to burnout in their careers, collusions between team members to support decisions with unethical aspects, a tendency to redefine unethical behavior as acceptable and a lack of communication among team members.^(^^22^^)^

It is necessary, then, to support professionals as moral agents and to promote moral courage for the development of ethical practices in our pediatric ICUs. Strategies for this include educating professionals about codes of conduct and professional ethics for the development of moral competence, assisting professionals in identifying and managing risks inherent to chosen behaviors, recognizing situations that trigger aggressive behaviors and generate fear and optimizing communication among team members in order to stimulate negotiation and reduce tensions resulting from disagreements and conflicts.^(^^23^^)^

### Confronting moral distress and promoting moral courage and resilience - "in our team, everyone has a voice"

Recent research demonstrates ways to overcome the reality of moral distress from an approach that addresses organizational, administrative and personal issues. In this sense, the management of the workload, distribution of resources, support for good relationships with managers, implementation of adequate labor policies, provision of a policy of openness to divergent opinions and free thinking and appreciation for the multidisciplinary team are influential organizational factors in the ethical work environment.^(^^24^^)^

Organizational strategies to improve the work environment and minimize moral distress include formal and informal discussions of ethical dilemmas experienced by team members (debriefings or "broadcasting sessions"), ethics committee consultations (to provide support and clarification for the ethical dilemma in question) and rounds, or visits, to discuss cases with ethical dilemmas in an anticipatory way (before a flare-up).

Other managerial strategies aimed at professionals include cultivating sensitive intentions, that is, encouraging professionals to engage in practices that promote kindness, generosity and humanism.

It is also necessary for administrators to recognize the phenomenon of personal suffering so that moral distress can be perceived by professionals as something that is "permitted," a sign of humanity and an affirmation of moral values. Institutions should promote techniques and offer courses and seminars that stimulate reflection and questioning in the practice of the health professional to help the professional differentiate himself or herself from the patient, that is, to not suffer for the suffering of the other. Such reflections should stimulate professional insight, that is, motivate professionals not to overestimate their identification with the patient's situation to help them avoid negative emotions. Still, one must cultivate the moral sensitivity of professionals, encouraging them to trust their intentions and monitor the emotions that can trigger moral distress. Pediatric ICU facilities should promote and refine ethical reasoning through continuing education so practitioners have good definitions of what is ethically acceptable (e.g., the concept of brain death and the removal of life support as an acceptable measure) in their environments.^(^^25^^)^

From a personal point of view, the importance of providing team members with an environment in which everyone has their voice heard without fear of reprisals or criticism (openness policy) is important, as is recognizing divergences of opinions according to different professional experiences and maintaining the patient and what is best for him or her as the main focus*.* The strengthening of relationships, respect for teamwork and minimizing of hierarchies ("everyone here has a voice") are fundamental to the development of an ethical and healthy practice.^(^^24^^)^

Methods for managing moral distress also include the emotional states of balance and resilience. Resilience is defined as "the ability of an individual to respond to stress in a healthy and adaptive way so that personal goals are achieved with minimal psychological and physical costs."^(^^13^^)^ In this context, authors suggest, at the personal level, creating zones of resilience as a strategy-for example, taking a reflexive pause before meeting with a family (such as when informing them that a prognosis is very bad), reflecting on the intentionality of an act, enabling mutual support between professionals of the same or different types ("I need to tell you what happened to me"), promoting the realization of a balanced practice that includes training the mind to avoid distractions and emotional confusion (mindfulness) and the monitoring of somatic responses, i.e., the recognition of bodily responses to difficult situations faced by professionals (tachycardia, palpitations, rapid breathing and headaches). Consequently, professionals involved in the care of severely ill children should take care of their own health by getting sufficient sleep, eating a balanced diet, performing physical exercise and cultivating interests beyond work such as hobbies. These techniques can contribute to a sense of stability.^(^^25^^)^ Table 2 is a summary of suggestions for coping with moral distress.

**Table 2:** Strategies for coping with moral distress at the organizational, personal and administrative levels

Organizational strategies	Personal strategies	Administrative strategies
Workload management and resource distribution, support for good relationships with managers, implementation of appropriate labor policies, provision of a policy of openness to differences of opinions and free thinking, valuation of the multidisciplinary team	Creating areas of resilience, such as a reflexive pause before an encounter with a patient to reflect on the intentionality of the act	Cultivating sensitive intentions, that is, encouraging professionals to engage in practices that privilege kindness, generosity and humanism
Formal and informal discussions of ethical dilemmas experienced by team members (debriefings or "broadcasting sessions")	Mutual support between professionals from the same or different categories	Recognition of the phenomenon of personal suffering so that moral distress can be perceived by the professional
Formal ethical consultancies to provide support and clarification on the ethical dilemma in question	Conduct a cautious practice, which includes training the mind to avoid distractions and emotional confusion	Providing techniques for cultivating reflection and questioning during practice to help the professional to differentiate himself from the patient by stimulating insight, that is, to help the professional not to overestimate his identification with the patient's situation in order to avoid negative emotions
Rounds or visits to discuss cases with ethical dilemmas present in daily practice in an anticipatory way (before the flare-up)	Monitoring somatic responses through the recognition of bodily responses to difficult situations faced by professionals	Cultivating the moral sensitivity of professionals, encouraging them to trust their intentions and monitoring of emotions that can trigger moral distress

### Integrating practice and research for understanding moral distress in pediatrics

Pediatric ethics research (which can be extended to adult ICU situations where the patient cannot communicate or has no family members) provides an important source for the development of practices aimed at the patient's best interests by promoting agency and moral courage and addressing and preventing the onset of moral distress. Through research and clinical studies, the concept of "best interests" can be improved. Important areas for further research include how to improve communication with families, mature children and/or young people who can make decisions about their treatments; how to improve communication, interprofessional and multidisciplinary collaboration through the creation of ethical standards for care; and how to promote respect for local cultures, among others.

In this context, interdisciplinary research on pediatric ethics plays a fundamental role in solidifying the multiprofessional approach. The international approach, bringing different contexts and cultural and social experiences together, can also contribute to a broader understanding of ethical issues related to care, helping establish the concept of best interests within the local sociocultural reality.^(^^26^^)^ Over time, the best preparation for different professionals who will face these situations in their work should be a constant target of research.^(^^26^^)^

## FINAL COMMENTS

Moral distress is an inherent reality of practice in intensive care units, a phenomenon that is still underexplored in the national literature. It is suggested that, in order to identify and discuss the extent of moral distress in clinical practice, health professionals should be recognized as moral agents who should understand and stimulate the development of their own moral courage. Moral courage is required when advocating for a patient, focusing on him or her, and when facing conflicts with colleagues or families. Work environments and organizational issues need to be addressed in order to encourage team members to overcome fears and to avoid intemperate personal attitudes when faced with situations that they consider unethical and contrary to their perception of what is right or wrong. There is a need to identify and challenge institutional barriers in order to establish an ethical environment and an attitude of mutual respect among professionals in the work environment. Furthermore, there is a need to stimulate and integrate research with daily clinical practice regarding the establishment of ethical standards so that conduct is in accordance with the best evidence available within the local socioeconomic context.

The goal should not be to eliminate moral distress, since health professionals are human and have values, but to minimize it, so that care for the seriously ill child continues to be done with professionalism and mutual respect. Only then will health professionals' careers be sustainable.
